# Identification and validation of aging-related gene signatures and their immune landscape in diabetic nephropathy

**DOI:** 10.3389/fmed.2023.1158166

**Published:** 2023-06-19

**Authors:** Yingchao Liang, Zhiyi Liang, Jinxian Huang, Mingjie Jia, Deliang Liu, Pengxiang Zhang, Zebin Fang, Xinyu Hu, Huilin Li

**Affiliations:** ^1^Graduate School of Guangzhou University of Traditional Chinese Medicine, Guangzhou, China; ^2^The Fourth Clinical Medical College of Guangzhou University of Chinese Medicine, Shenzhen, China; ^3^Guangdong Provincial Hospital of Integrated Traditional Chinese and Western Medicine Affiliated to Guangzhou University of Chinese Medicine, Foshan, China; ^4^Department of Endocrinology, Shenzhen Traditional Chinese Medicine Hospital, Shenzhen, China

**Keywords:** diabetic nephropathy, SVM-RFE, random forest, GHR, VEGFA, EFEMP1, gene, aging

## Abstract

**Background:**

Aging and immune infiltration have essential role in the physiopathological mechanisms of diabetic nephropathy (DN), but their relationship has not been systematically elucidated. We identified aging-related characteristic genes in DN and explored their immune landscape.

**Methods:**

Four datasets from the Gene Expression Omnibus (GEO) database were screened for exploration and validation. Functional and pathway analysis was performed using Gene Set Enrichment Analysis (GSEA). Characteristic genes were obtained using a combination of Random Forest (RF) and Support Vector Machine Recursive Feature Elimination (SVM-RFE) algorithm. We evaluated and validated the diagnostic performance of the characteristic genes using receiver operating characteristic (ROC) curve, and the expression pattern of the characteristic genes was evaluated and validated. Single-Sample Gene Set Enrichment Analysis (ssGSEA) was adopted to assess immune cell infiltration in samples. Based on the TarBase database and the JASPAR repository, potential microRNAs and transcription factors were predicted to further elucidate the molecular regulatory mechanisms of the characteristic genes.

**Results:**

A total of 14 differentially expressed genes related to aging were obtained, of which 10 were up-regulated and 4 were down-regulated. Models were constructed by the RF and SVM-RFE algorithms, contracted to three signature genes: EGF-containing fibulin-like extracellular matrix (EFEMP1), Growth hormone receptor (GHR), and Vascular endothelial growth factor A (VEGFA). The three genes showed good efficacy in three tested cohorts and consistent expression patterns in the glomerular test cohorts. Most immune cells were more infiltrated in the DN samples compared to the controls, and there was a negative correlation between the characteristic genes and most immune cell infiltration. 24 microRNAs were involved in the transcriptional regulation of multiple genes simultaneously, and Endothelial transcription factor GATA-2 (GATA2) had a potential regulatory effect on both GHR and VEGFA.

**Conclusion:**

We identified a novel aging-related signature allowing assessment of diagnosis for DN patients, and further can be used to predict immune infiltration sensitivity.

## 1. Introduction

Diabetic nephropathy (DN) is one of the most severe microangiopathic complications of diabetes mellitus, and it may be considered the significant cause of death in patients with end-stage renal disease (ESRD) (1). The significant pathological alterations in DN include glomerulopathy, renal tubular atrophy and renal interstitial fibrosis. According to an epidemiological survey, DN accounts for 47% of ESKD patients in the US and up to 60% in countries such as Malaysia and Singapore (2). In an autopsy study, histologically confirmed DN’s prevalence was higher than clinically diagnosed (3). Due to the widespread prevalence and poor prognosis of diabetic nephropathy, it is critical to further explore the pathogenesis of diabetic nephropathy and potential biomarkers for early identification and prevention of diabetic nephropathy.

Cellular senescence is the state in which the G1 and G2 phase of the cell cycle comes to an infinite halt, and it plays a crucial role in renal aging and diseases. The kidney with DN displays an accelerated senescent phenotype in tubule cells and podocytes (4), and high glucose can cause premature senescence in human glomerular mesangial cells and proximal tubule epithelial cells (5, 6). The detrimental effects of persistent cellular senescence are closely related to the senescence-associated secretory phenotype (SASP), which is characterized by multiple factors that contribute to chronic inflammation, adverse tissue remodeling, and fibrosis (7). There is a strong positive association between age and nephrosclerosis in healthy adults (8), and epithelial cell cycle arrest in G2/M mediates kidney fibrosis after injury (9). Kidney fibrosis is one of major outcome of diabetic nephropathy, and the functional consequence of the epithelial-to-mesenchymal transition (EMT) program during fibrotic injury is an arrest in the G2 phase of the cell cycle (10). Cellular phenotypic transitions play an important role in the development of diabetic nephropathy. Macrophage-myofibroblast transition (MMT) can lead to progressive renal fibrosis induced by ongoing chronic inflammation (11).

Chronic inflammation has become one of the essential hallmarks driving aging (12). Aging T cells accelerate renal fibrosis in mice and maintain a pro-inflammatory state (13). M1 macrophages secrete pro-inflammatory cytokines to promote tissue inflammation and kidney injury (14). Increasing researches suggest a central role for chronic inflammation and immune cell infiltration in DN. A significant increase in macrophages has been observed in the glomerular and tubular interstitium of human T2D (15), and aberrant intrarenal infiltration and the activation of T cells in interstitium are the underlying immunopathological mechanisms of diabetic kidney injury (16). In another experiment, T cell immunoglobulin domain and mucin domain-3 (Tim-3) has been found to play a critical role in developing DN in macrophages (17). Cellular phenotypic transformations such as MMT and EMT are involved in immune inflammatory pathways promoting the development of DN. A growing body of research provides important clues to the relationship between ageing and immunity, but at present there is a lack of concrete explanation between them and the mechanisms underlying their role in DN.

MicroRNAs (miRNAs) have been identified as epigenetic factors with the ability to influence aging and DN (18, 19). miR-21 targets Smad7/TGF-β1 pathways to aggravate EMT and accelerate renal fibrosis in diabetic mice (20). High expression of miR-let-7c can reduce renal fibrosis in rats and promote the resolution of inflammation (21). In addition, some researches have shown that transcription factors(TFs) play an essential role in the pathogenesis and outcome of DN (22, 23). Currently, many DN therapeutic drug mechanisms involve renal aging and immune response. The dipeptidyl peptidase-4 (DPP-4) inhibitor, sodium-glucose co-transporter 2 (SGLT-2) inhibitors and Rho-associated kinase (ROCK) inhibitor can ameliorate diabetic renal fibrosis and suppress proinflammatory pathways, thereby delaying the progression of DN (24–27). AcSDKP alone or in combination with ACE inhibitors can prevent renal fibrosis by inhibiting the EndMT program in the kidneys of diabetic mice (28). Glycolysis inhibitors decrease the expression of both inflammatory and fibrotic genes by suppressing glycolytic activation in macrophages (29). Therefore, it is instructive to explore the mechanisms of diabetic nephropathy based on senescence action, which can help identify clinically significant aging genes and provide potential biomarkers for diagnosis and treatment.

Our research aims to identify aging-related gene signatures and their immune landscape. We adopted a combination of the RF and the SVM-RFE model to screen for characteristic genes and validate the diagnostic efficacy and expression patterns in three independent test cohorts. In addition, we further explored and validated the association of characteristic genes with immune infiltration in DN samples. We also predicted potential miRNAs and TFs further to elucidate the molecular regulatory mechanisms of the signature genes. The findings may provide a novel theoretical foundation for developing diagnostic biomarkers and therapeutic targets of diabetic nephropathy based on aging-related genes.

## 2. Materials and methods

### 2.1. Data collection and processing

Four expressions profiling by array about DN were obtained from the Gene Expression Omnibus (GEO) repository.^1^ GSE30122 contains 19 DN and 50 healthy control (HC) samples, where DN and control samples derived from glomerular tissues are used as a training cohort, and its platform number is GPL571. GSE96804(DN = 41, HC = 20, GPL17586), GSE1009(DN = 3, HC = 3, GPL8300), GSE30529(DN = 10, HC = 12, GPL571) were mainly used for test and validation. GSE96804 and GSE1009 were obtained from glomerular biopsy specimens, and GSE30529 was obtained from tubular biopsy specimens. Probe annotation was performed using R software to obtain mRNA-seq arrays containing gene symbols. Probes with multiple genes were excluded, and the mean of the corresponding genes was calculated. Genes with multiple probes were calculated as averages. The data were normalized *via* the “normalize between arrays” function of the R package “limma” to achieve consistency between arrays. Differentially expressed genes (DEGs) were screened in the DN and control samples with the criteria of |log2 fold change| ≥ 1.0 and Pvalue < 0.05. 307 aging-related genes (30) were selected from Human Aging Genomic Resources^2^ (Supplementary Table 1). Mapping aging-related genes in DEGs to obtain aging-related DEGs, visualized by R package “ggVennDiagram.”

### 2.2. Functional enrichment analysis

Functional enrichment analysis was conducted to identify the corresponding biological pathways involved in the aging-related DEGs. Gene ontology (GO) enrichment analysis interpreted the biological significance of genes from three perspectives: biological process (BP), cellular component (CC), and molecular function (MF). Kyoto Encyclopedia of Genes and Genomes (KEGG) pathway analysis was used to assess gene function systematically. R package “Clusterprofiler” was employed to automate the enrichment analysis of gene clusters. 10 BP terms, 5 MF terms, 5 CC terms, and 20 KEGG terms with the criteria of value of *p* < 0.01 and *q*-value < 0.05 were visualized by R package “ggplot2” and “enrichplot.” R package “org.Hs.eg.db” was used for conversion between gene IDs.

### 2.3. Gene set enrichment analysis

Gene set enrichment analysis (GSEA) was performed in the DN and the control groups. The top terms of HALLMARK pathways with significant enrichment results were exhibited based on Net Enrichment Score (NES), gene ratio, and *p* value. Gene sets with |NES| > 1, Pvalue < 0.01, and *Q* value < 0 0.05 were considered to be enrichment significant (31). The gene set database (h.all.v2022.1.Hs.symbols) used during the analysis was downloaded from the Molecular Signature Database.^3^

### 2.4. Construction of machine learning models

Random Forest (RF) model and Support Vector Machine Recursive Feature Elimination (SVM-RFE) model were established on the ground of the training cohort. RF is a classification algorithm that uses multiple trees to train and predict samples and is characterized by high accuracy. The max number of trees in the RF model was set to 500, and the mean decrease gini (MDG) was used as an essential measure (32). MDG involved in the random forest algorithm provides ways to quantify which indices contribute most to classification accuracy. Based on the RF model established by R package “randomForest,” we analyzed the significance of variables and selected critical genes with MDG > 1. Furthermore, a machine learning technique, SVM-RFE, was utilized to find the best variable by deleting the feature vector generated by SVM (33). R package “e1071” was adopted to further screen aging-related genes in DN. The intersections contracted by the RF and SVM-RFE models were considered characteristic genes and used for subsequent analysis.

### 2.5. Establishment of a nomogram

To predict the occurrence of DN based on aging-related genes, characteristic genes were incorporated to establish a nomogram using R package “rms.” A receiver operating characteristic (ROC) curve was drawn to assess the diagnostic ability of the nomogram model. The calibration curve was utilized for evaluating the accuracy of the nomogram.

### 2.6. Validation of characteristic genes in test cohorts

We used the training cohort to evaluate the diagnostic performance of the characteristic genes, and three independent cohorts (GSE1009, GSE96804, GSE30529) were used to validate. ROC curve analyses were conducted with the R package “pROC,” The area under the ROC curve (AUC) values were utilized to assess the predictive utility of characteristic genes. A 95% Confidence interval (CI) was employed to estimate the range of the parameters. Furthermore, GSE30529 was used to verify the diagnostic performance of characteristic genes in renal tubules of DN patients. In addition, expression patterns of characteristic genes were validated in test cohorts by comparing the samples of control and patients with DN.

### 2.7. The Single-Sample Gene Set Enrichment Analysis

The Single-Sample Gene Set Enrichment Analysis (ssGSEA) algorithm was adopted to assess the relative abundance of infiltration in our study (34). R package “GSEA” was used to quantify different immune cell enrichment scores with Gaussian parameters. ssGSEA algorithm is a rank-based method that defines a score representing the degree of absolute enrichment of a particular gene set in each sample. The gene sets from published studies were fed into the ssGSEA algorithm for enrichment analysis, and the gene set for marking each immune cell was obtained from the study of Dr. Charoentong (35). The ssGSEA scores of 28 immune cells were correlated, and the results were visualized by R package “corrplot,” and the results with Pvalue < 0.05 were considered significant. Spearman correlation coefficient was calculated to measure the correlation between the expression of characteristic genes and immune cell infiltration abundance.

### 2.8. Construction of the molecular interaction networks

We created the protein–protein interaction (PPI) network to acquire insights into cellular machinery operations for aging-related genes through the STRING database^4^. The minimum required interaction score was set as medium confidence (0.400).

To explore the molecular and regulatory mechanisms of the characteristic genes, we tried to demonstrate the regulatory transcription factors (TFs) and miRNAs in a network-based approach. TFs were identified from the JASPAR repository on the NetworkAnalyst platform^5^, and miRNAs targeting characteristic genes were identified similarly based on the TarBase database. The interaction network about the relationship between genes and miRNAs, genes, and TFs was constructed by Cytoscape (V3.9.1) software.

### 2.9. Association between characteristic genes and clinical features

The Nephroseq V5 tool^6^ was employed to validate the expression of the characteristic genes in diabetic nephropathy and other kidney-related diseases. Correlation analysis and subgroup analysis between the characteristic genes and clinical features were also performed to verify the potential functions of aging-related genes in DN. We acquired the raw data from the Nephroseq V5 database and utilized R package “ggpubr” to redraw the scatter plots.

### 2.10. Statistical analysis

All statistical tests were implemented utilizing R software 4.2.1. The Wilcoxon rank sum test was used to analyze the significance of differential gene expression in the GEO datasets. Student’s t-test was utilized for analyzing continuous variables between the two groups. Comparisons between multiple groups were conducted with ANOVA test. The correlation between the variables was determined using Spearman’s correlation test. All statistical *p*-values were two-sided, and *p* < 0.05 was considered statistical significance.

## 3. Results

### 3.1. Identification of aging-related DEGs

**Figure 1:**
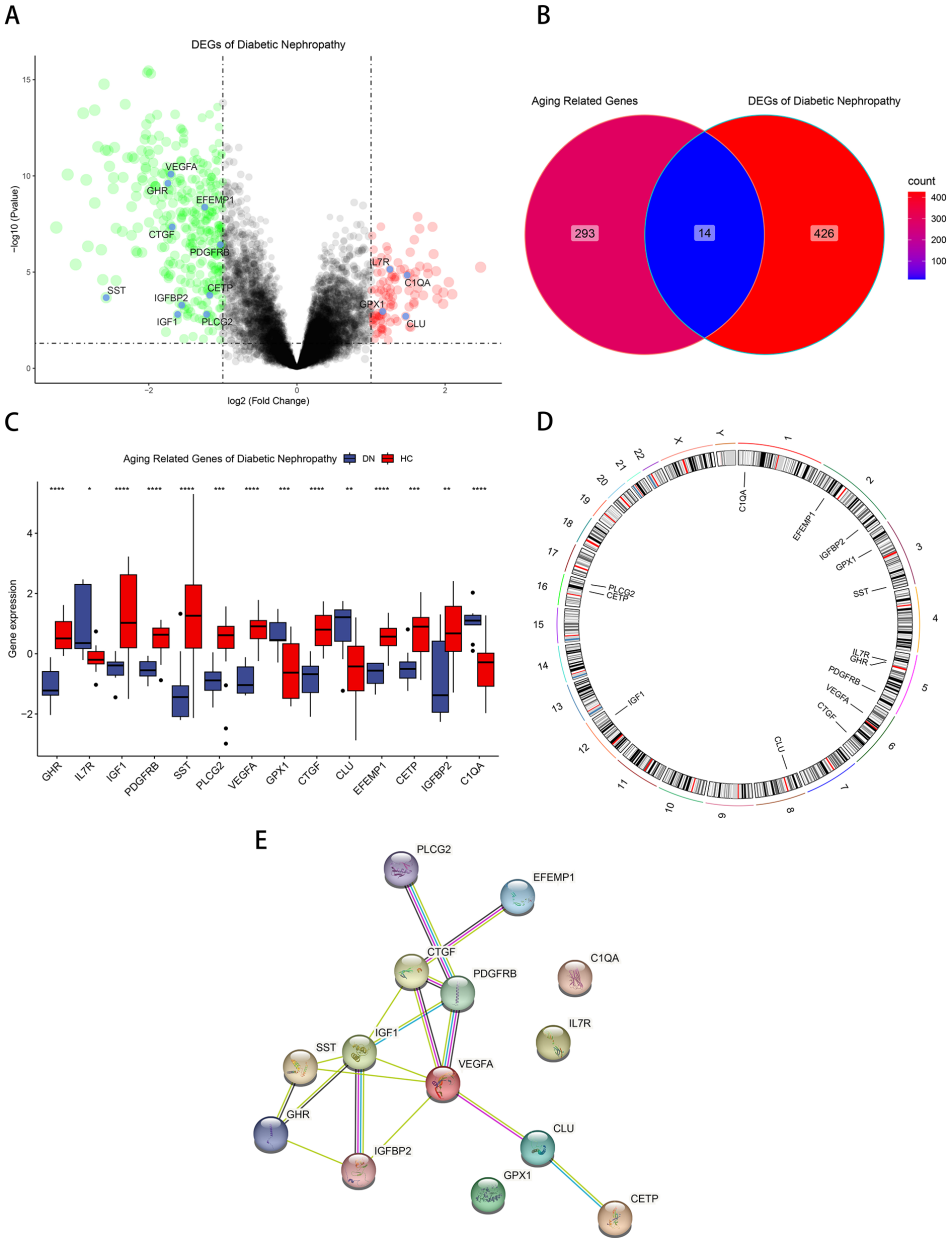
Identification of aging-related DEGs. **(A)** Volcano plot of DEGs between normal and DN samples in diabetic nephropathy. **(B)**Venn diagram of aging-related genes and DEGs of DN. **(C)** Box plot of 14 aging-related DEGs in DN and healthy samples. ^*^p < 0.05; ^**^p < 0.01; ^***^p < 0.001; ^****^p < 0.0001. **(D)** The specific location of each aging-related DEGs on the chromosome. **(E)**The PPI network revealed the interactions among the aging-related DEGs.

Differential gene analysis was performed in the glomerular samples of the training cohort GSE30122 with Pvalue < 0.05 and absolute value of log2 fold change > 1. Compared with healthy controls, DN patients had 334 downregulated DEGs and 106 upregulated DEGs (Figure 1A). Fourteen aging-related DEGs were obtained by mapping DEGs to aging-related genes (Figure 1B), a box plot highlighted their expression patterns in DN samples and healthy controls (Figure 1C). Growth hormone receptor (GHR), insulin-like growth factor I (IGF1), insulin-like growth factor-binding protein 2 (IGFBP2), vascular endothelial growth factor A (VEGFA), platelet-derived growth factor receptor beta (PDGFRB), somatostatin (SST), 1-phosphatidylinositol 4,5-bisphosphate phosphodiesterase gamma-2 (PLCG2), CCN family member 2 (CTGF), EGF-containing fibulin-like extracellular matrix (EFEMP1), cholesteryl ester transfer protein (CETP) had lower expressions in DN, and interleukin-7 receptor subunit alpha (IL7R), complement C1q subcomponent subunit A (C1QA), glutathione peroxidase 1 (GPX1), clusterin (CLU) had higher expressions in DN. The distribution of 14 genes in chromosomes was illustrated in Figure 1D. A PPI network of aging-related DEGs in DN was constructed using the STRING database. The network included 17 edges and 14 nodes (Figure 1E), VEGFA and IGF1 had the tightest interaction (Degree = 6) with other nodes. Three up-regulated genes, IL7R, C1QA, GPX1, did not have connection to other notes.

### 3.2. Functional and pathway enrichment analysis of aging-related DEGs

**Figure 2:**
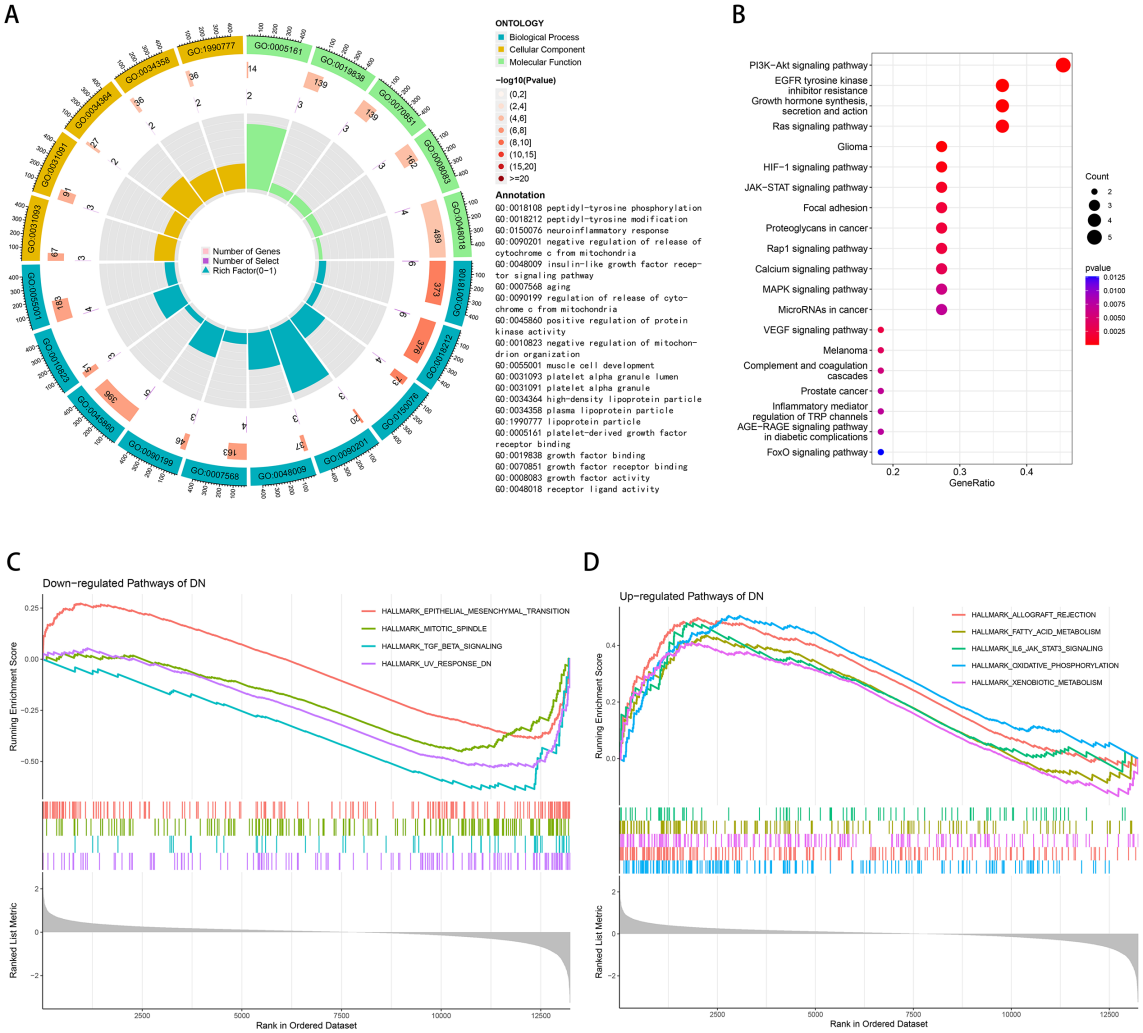
Functional and pathway enrichment analysis. **(A)** GO terms in the enrichment analysis of aging-related DEGs. **(B)** KEGG terms in the enrichment analysis of aging-related DEGs. **(C)** Down-regulated pathways of DN in the GSEA analysis. **(D)** Up-regulated pathways of DN in the GSEA analysis.

GO and KEGG enrichment analyses were performed to determine the potential biological functions of aging-related DEGs. The most significant enrichment terms for GO were peptidyl-tyrosine modification, neuroinflammatory response, negative regulation of release of cytochrome c from mitochondria, insulin-like growth factor receptor signaling pathway, aging, positive regulation of protein kinase activity, negative regulation of mitochondrion organization, muscle cell development, growth factor binding, growth factor activity, etc. (Figure 2A). It is primarily involved in biological processes such as aging, energy metabolism and growth (36–38). Neuroinflammatory response implies that these genes may be involved in immune regulation, which can be further explored. The KEGG pathway is mainly enriched in PI3K-Akt signaling pathway, EGFR tyrosine kinase inhibitor resistance, growth hormone synthesis, secretion and action, Ras signaling pathway, HIF-1 signaling pathway, JAK–STAT signaling pathway, Rap1 signaling pathway, calcium signaling pathway, MAPK signaling pathway, VEGF signaling pathway, complement and coagulation cascades, inflammatory mediator regulation of TRP channels, AGE-RAGE signaling pathway in diabetic complications, FoxO signaling pathway (Figure 2B). According to GSEA results, TGF-beta signaling, UV response DN, mitotic spindle, epithelial mesenchymal transition was down-regulated in DN (Figure 2C) and xenobiotic metabolism, fatty acid metabolism, IL6-JAK-STAT3 signaling, allograft rejection, oxidative phosphorylation was up-regulated in DN (Figure 2D).

### 3.3. Identification and evaluation of characteristic genes

**Figure 3:**
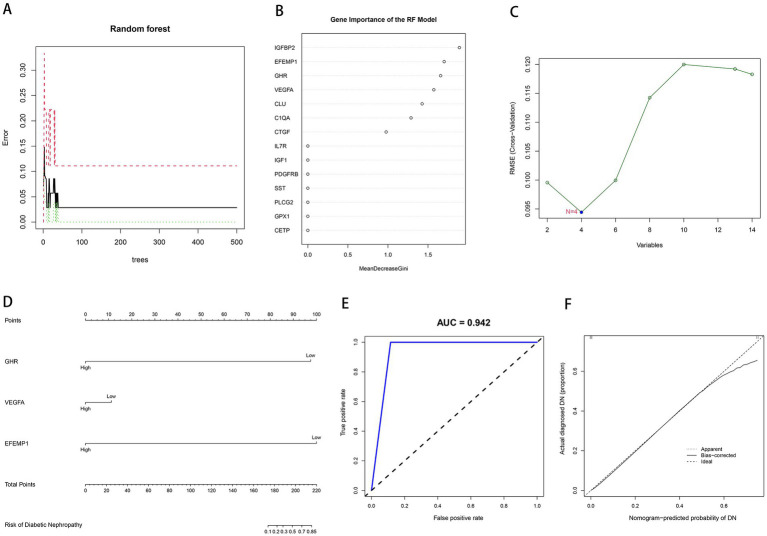
Construction of machine learning models. **(A)** The influence of the number of decision trees on the error rate. The *x*-axis represents the number of decision trees, and the *y*-axis indicates the error rate. **(B)** Results of the Gini coefficient method in the RF model. The *x*-axis represents the MDG, and the *y*-axis indicates the genetic variable. **(C)** Support Vector Machine Recursive Feature Elimination (SVM-RFE) algorithm to screen candidate genes. The x-axis indicates the genetic variable, and the y-axis represents RMSE cross-validated. **(D)** Nomogram for the diagnostic model of DN. **(E)** Receiver operating characteristic (ROC) curve for the diagnostic model. **(F)** Calibration curve for the diagnostic model.

We input aging-related DEGs into the random forest model. Referring to the relationship plot between the model error and the number of decision trees (Figure 3A), 9 trees were selected as the final model parameter, indicating that the model error is minimum and stable. In constructing the random forest model, we measured the variable importance of the output results (Gini coefficient method) in decreasing accuracy and decreasing mean square error (39). Six genes with MDG greater than 1 were then identified as candidate genes. Among these six variables, IGFBP2 and EFEMP1 were the most important, followed by GHR, VEGFA, CLU, and C1QA (Figure 3B). Four candidate genes, GHR, VEGFA, CTGF and EFEMP1, were extracted from aging-related DEGs by SVM-RFE algorithm (Figure 3C) because of the minimum root mean square error (RMSE). Candidate genes from two models were overlapped, and 3 aging-related characteristic genes in DN were identified: EFEMP1, GHR, and VEGFA. A diagnostic model was constructed by logistic regression based on expressions of the characteristic genes in the training cohort and visualized as a nomogram (Figure 3D). The model showed a high AUC value (0.942), confirming the excellent predictive performance (Figure 3E). The calibration plot evaluated the bias of the prediction model with the actual event (Figure 3F).

### 3.4. ROC curves of characteristic genes in the evaluation and validation cohorts

**Figure 4:**
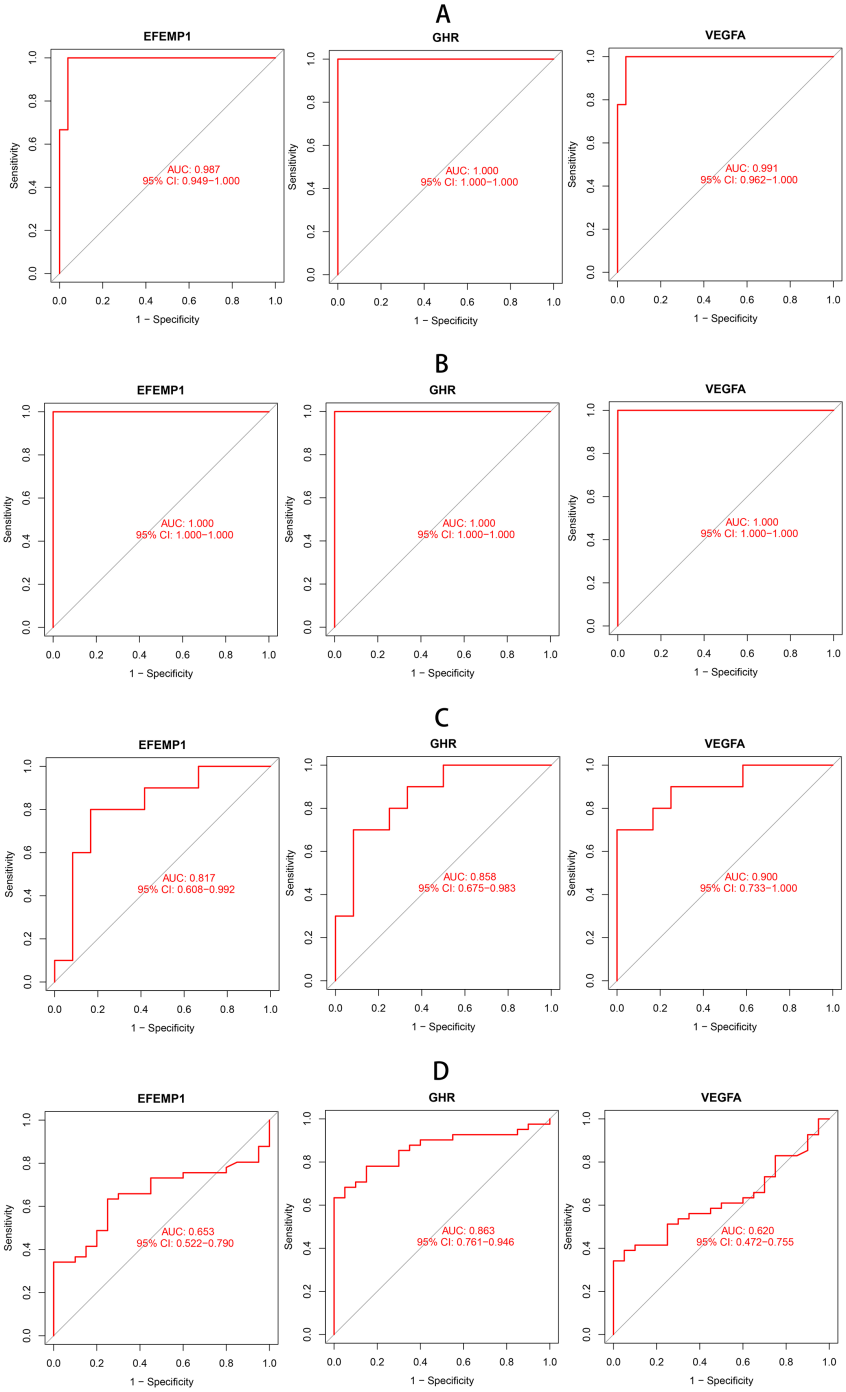
Receiver operating characteristic (ROC) curves of aging-related characteristic genes in DN. **(A)** ROC curves of three characteristic genes in the training cohort. **(B)** ROC curves of three characteristic genes in the test cohort GSE1009. **(C)** ROC curves of three characteristic genes in the test cohort GSE30529. **(D)** ROC curves of three characteristic genes in the test cohort GSE96804.

To estimate the predictive utility of the characteristic genes, we performed a ROC curve analysis and validated it in three independent cohorts. All three characteristic genes illustrated a remarkably distinguishing efficiency. AUC combines sensitivity and specificity and can authenticate the inherent validity of a diagnostic test. AUC values of EFEMP1, GHR and VEGFA were 0.987 (95% CI: 0.949–1.000), 1.000 (95% CI: 1.000–1.000) and 0.991 (95% CI: 0.962–1.000) in the DN training cohort, respectively (Figure 4A). The characteristic genes consistently showed excellent diagnostic performance in three independent test cohorts. AUC values of three characteristic genes were all 1.000 (95% CI: 1.000–1.000) in GSE1009 (Figure 4B). In GSE30529, AUC values of EFEMP1, GHR and VEGFA were 0.817 (95% CI: 0.608–0.992), 0.858(95% CI: 0.675–0.983) and 0.900 (95% CI: 0.733–1.000; Figure 4C). GHR maintained a high AUC value of 0.863 (95% CI: 0.761–0.946) in GSE96804 (Figure 4D). These characteristic genes could be considered potential diagnostic biomarkers for DN.

### 3.5. Evaluation and validation of expression patterns of characteristic genes

**Figure 5:**
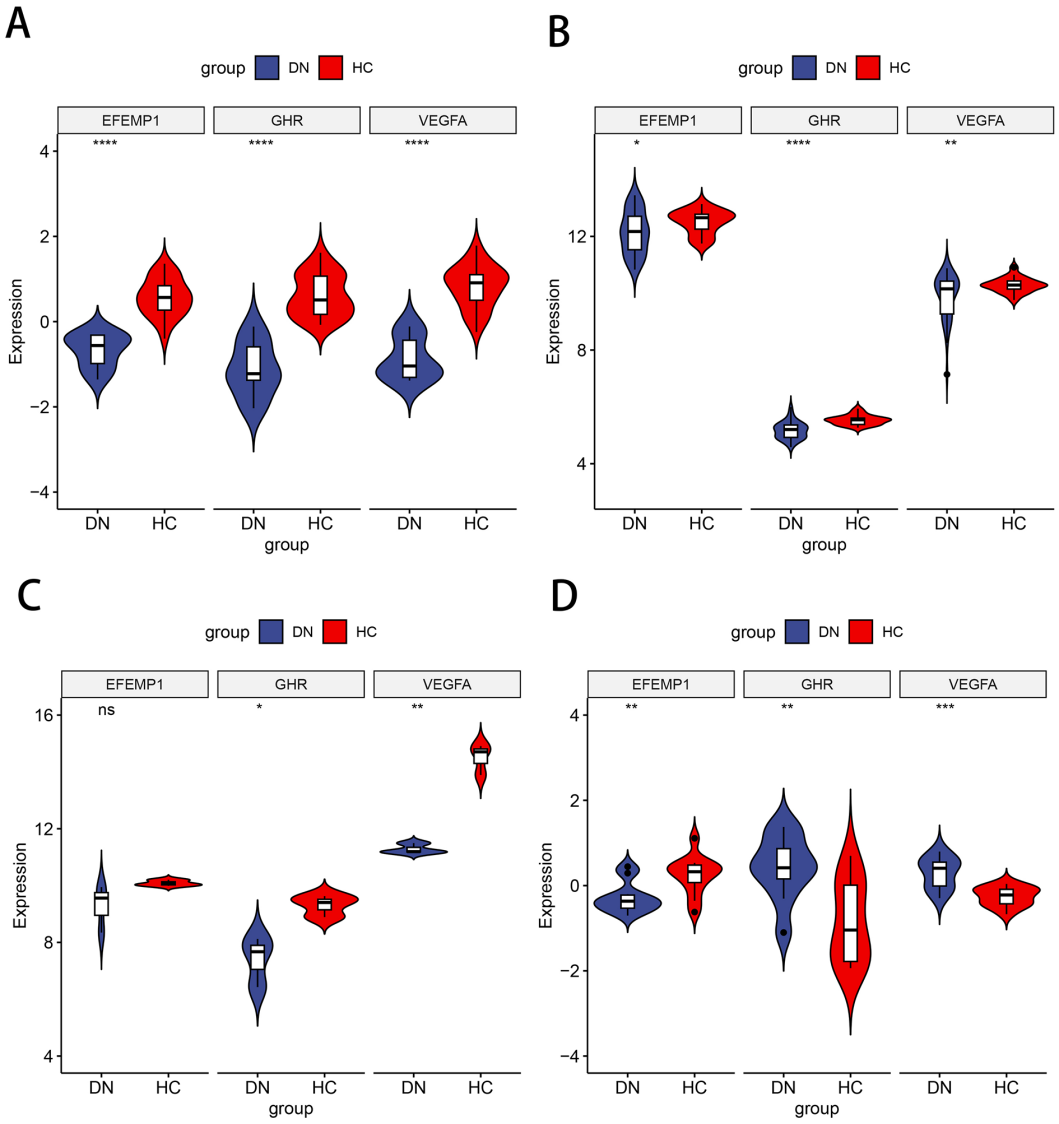
Expression patterns of the characteristic genes in the training and test cohorts. **(A)** Violin plot of expression about three characteristic genes in the training cohort. **(B)** Violin plot of expression about three characteristic genes in the test cohort GSE96804. **(C)** Violin plot of expression about three characteristic genes in the test cohort GSE1009. **(D)** Violin plot of expression about three characteristic genes in the test cohort GSE30529.

**Figure 6:**
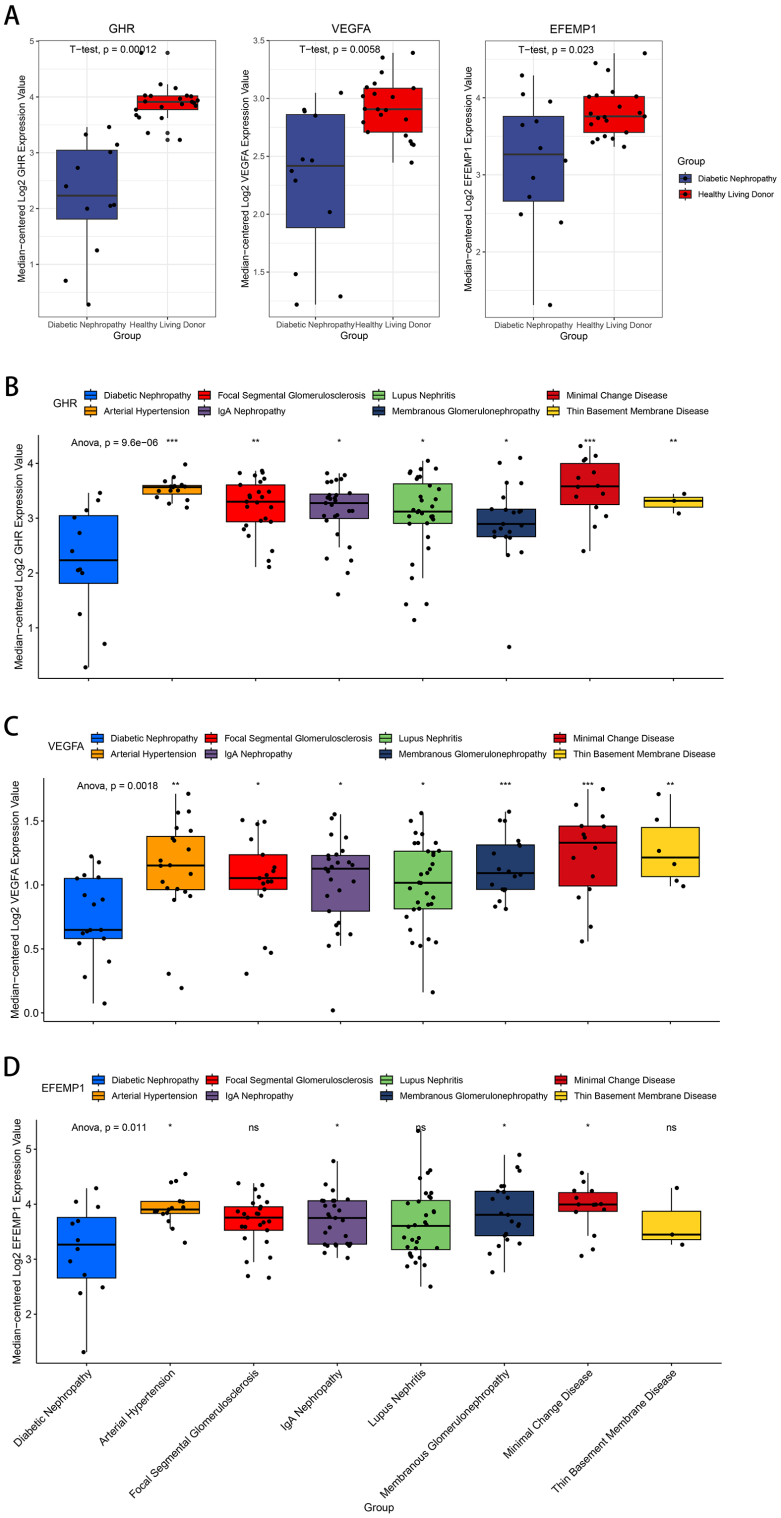
Expression patterns of the characteristic genes in the Nephroseq V5 database. **(A)** Box plots of the expression about three characteristic genes in the DN samples and healthy living donors. **(B–D)** Box plots of the expression about GHR **(B)**, VEGFA **(C)** and EFEMP1 **(D)** in multiple renal diseases.

Compared with the control group, decreased expressions (*p* < 0.0001) of EFEMP1, GHR and VEGFA were observed in the glomerular samples from the DN training cohort (Figure 5A). Test cohorts validated the results, and consistent gene expression patterns with statistical significance were obtained in GSE96804 (Figure 5B) and GSE1009 (Figure 5C). Interestingly, the expression of EFEMP1 was still significantly downregulated (*p* < 0.01) in renal tubule samples from the DN test cohort. In contrast, the expressions of GHR and VEGFA were upregulated (*p* < 0.01) in renal tubules (Figure 5D). Furthermore, we validated the expression patterns of the characteristic genes in the Nephroseq V5 database. Three genes remained down-regulated in the DN group (Figure 6A). Notably, the characteristic genes were significantly under-expressed in DN compared to other kidney-related diseases (Figures 6B–D).

### 3.6. Assessment of immune cell infiltration in DN

**Figure 7:**
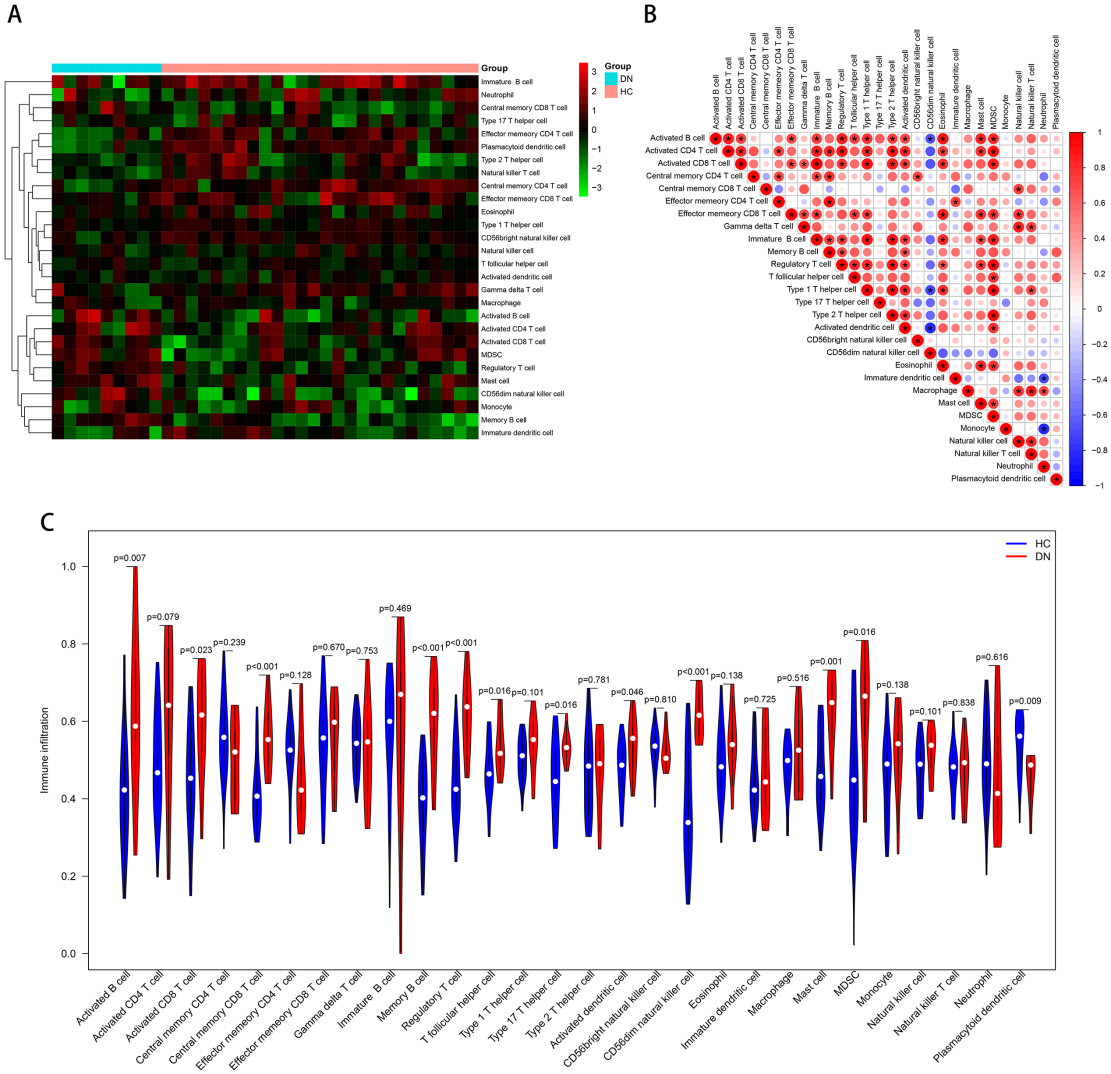
Immune cell infiltration analysis based on the ssGSEA algorithm. **(A)** The infiltration degree of 28 immune cells in each sample. Red squares indicate higher immune infiltration expression, and green squares indicate lower expression. **(B)** Correlation matrix of infiltration degree of immune cells in DN samples. **(C)** Violin plot of the differential analysis of each immune cell between two groups.

Based on the ssGSEA algorithm, we evaluated the immune infiltration of the training cohort. The level of infiltration of each immune cell in the DN and control samples is shown in Figure 7A. The infiltration between different immune cells was mostly positively correlated and statistically significant (Pvalue < 0.05; Figure 7B). On the contrary, CD56dim natural killer cells were negatively correlated with most other immune cells. Compared to the controls, most of the immune cells were highly expressed in DN: activated B cell (*P* value = 0.007), activated CD8 T cell (*P* value = 0.023), central memory CD8 T cell (*P* value < 0.001), memory B cell (Pvalue <0.001), regulatory T cell (*P* value <0.001), T follicular helper cell (*P* value = 0.016), type 17 T helper cell (*P* value = 0.016), activated dendritic cell (*P* value = 0.046), CD56dim natural killer cell (*P* value < 0.001), mast cell (*P* value = 0.001), MDSC (*P* value = 0.016). Only plasmacytoid dendritic cell (*P* value = 0.009) was more infiltrated in the control group (Figure 7C), and immune infiltration tended to be upregulated in DN samples. In the test cohort GSE96804, most immune cells were also more infiltrated in DN samples; Only neutrophil, monocyte and CD56dim natural killer cell were more infiltrated in the control group (Supplementary Figure 1).

### 3.7. Evaluation and validation of the associations between characteristic genes and immune landscape

**Figure 8:**
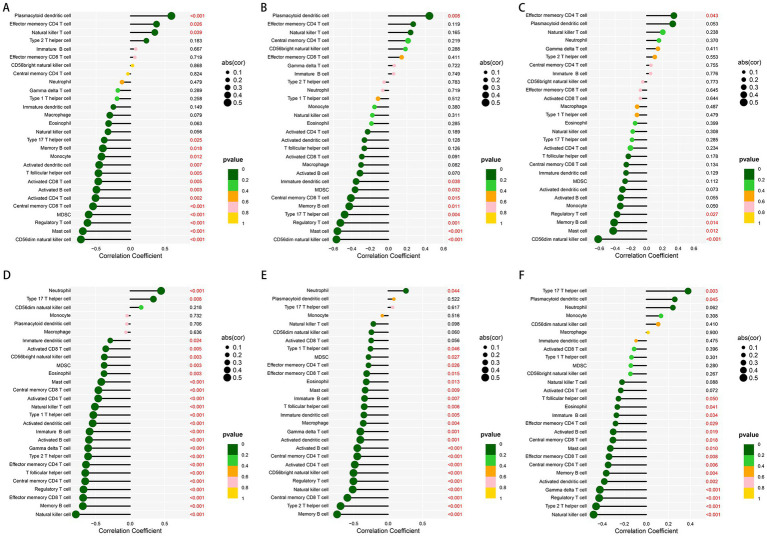
Correlation between the expression of three characteristic genes and different immune cell infiltrations in the training and test cohorts. **(A–C)** Lollipop plot of correlation between the expression of GHR **(A)**, VEGFA **(B)** and EFEMP **(C)** and immune infiltration in the training cohort. **(D–F)** Lollipop plot of correlation between the expression of GHR **(D)**, VEGFA **(E)** and EFEMP **(F)** and immune infiltration in the test cohort GSE96804.

In order to better understand the role of the characteristic genes in immune infiltration, we subsequently conducted spearman correlation analysis to ascertain whether these genes were correlated with immune cell infiltration. The expression of GHR and the infiltration of CD56dim natural killer cell, mast cell, regulatory T cell, MDSC, central memory CD8 T cell, activated CD4 T cell, activated B cells, activated CD8 T cell, T follicular helper cell, activated dendritic cell, monocyte, memory B cell, type 17 T helper cell were negatively correlated with statistical significance; plasmacytoid dendritic cell, natural killer T cell, and effector memory CD4 T cell were positively correlated with GHR (Figure 8A). The expression of VEGFA and EFEMP1 was also negatively correlated with the majority of immune cell infiltrates (Figures 8B,C), suggesting that the expression levels of the characteristic genes could reflect the immune landscape of DN patients. In the test cohort, we obtained similar validation results (Figures 8D–F), with an overall negative correlation between the expression of the characteristic genes and the levels of infiltration. T cells and B cells in the test cohort were significantly negatively correlated with the characteristic genes, while neutrophil was positively correlated. In addition, mast cell, regulatory T cell and memory B cell were significantly negatively correlated with the characteristic genes in both cohorts.

### 3.8. Prediction of TFs and microRNAs associated with characteristic genes

**Figure 9:**
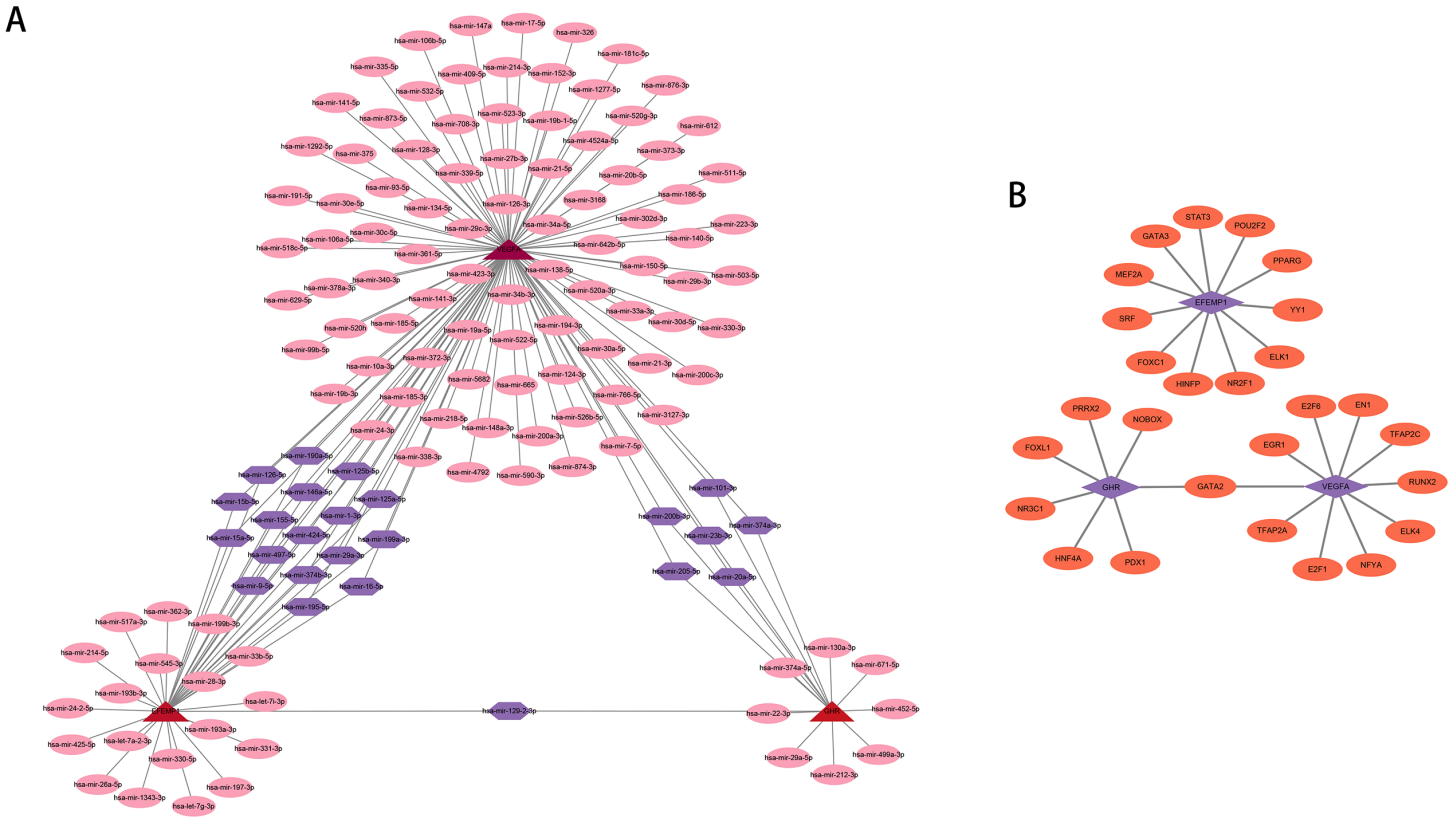
Potential regulatory molecules for the characteristic genes. **(A)** Three characteristic genes and their interactions with potential miRNAs based on the TarBase repository (142 nodes and 163 edges). The red nodes depict the characteristic genes, the pink nodes represent miRNAs associated with only one characteristic gene, and the purple nodes indicate miRNAs associated with multiple characteristic genes. **(B)** Three characteristic genes and their interactions with potential TFs based on the JASPAR database (30 nodes and 28 edges). The purple nodes depict the characteristic genes, and the orange nodes represent TFs associated with the characteristic genes.

We employed publicly available bioinformatic databases to reveal the potential changes and molecular regulatory mechanisms happening at the transcriptional level for the characteristic genes (Supplementary Table 2). miRNAs corresponding to the characteristic genes were predicted, and the mRNA-miRNA network based on the TarBase repository was constructed (Figure 9A); these genes were linked together by multiple shared miRNAs. There were 17 miRNAs associated with VEGFA and EFEMP1, 6 miRNAs associated with GHR and VEGFA, and 1 miRNA associated with GHR and EFEMP1. The potential TFs from the JASPAR database were acquired and the mRNA-TF network was developed (Figure 9B), and GATA2 has a regulatory effect on both GHR and VEGFA.

### 3.9. Clinical correlation of the characteristic genes with renal function

**Figure 10:**
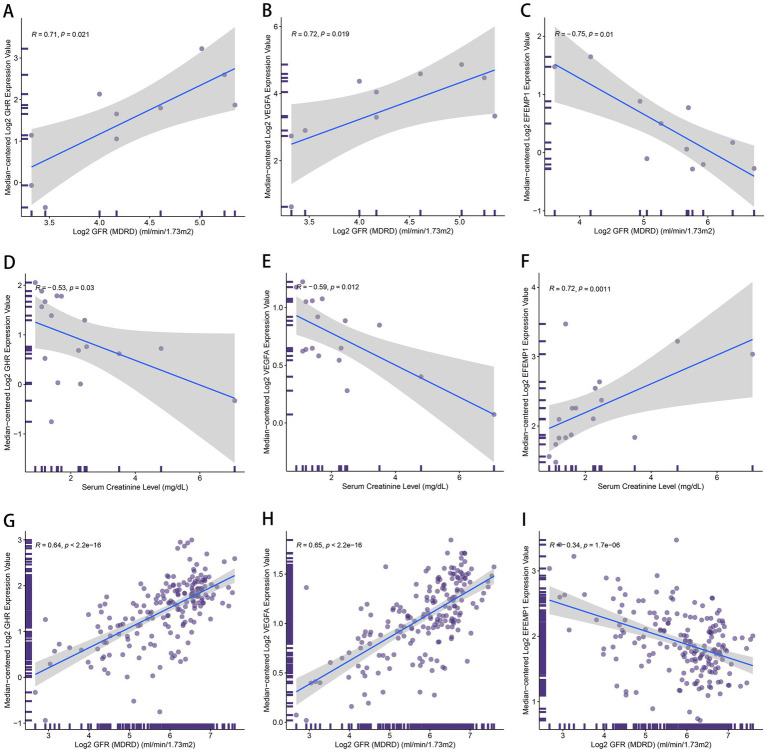
The correlation between the characteristic genes and clinical features. **(A–C)** Correlation plots about glomerular filtration rate (GFR) and the expression of GHR **(A)**, VEGFA **(B)** and EFEMP1 **(C)** in DN samples. **(D–F)** Correlation plots about serum creatinine level and the expression of GHR **(D)**, VEGFA **(E)** and EFEMP1 **(F)** in DN samples. **(G–I)** Correlation plots about GFR and the expression of GHR **(G)**, VEGFA **(H)** and EFEMP1 **(I)** in the samples with kidney-related diseases.

To further explicate the functions of these characteristic genes in DN, correlation analysis between 3 genes and clinical features was conducted based on the Nephroseq database. GHR (*r* = 0.71) and VEGFA (*r* = 0.72) demonstrated a robust positive correlation with GFR (Figures 10A,B), and GHR (*r* = −0.53) and VEGFA (*r* = −0.59) displayed a negative association with serum creatinine level (Figures 10D,E). Therefore, A higher expression of GHR and VEGFA may indicate better renal function in patients with DN, potentially providing a protective role against DN. EFEMP1 (*r* = −0.75) exhibited a firm negative correlation with GFR (Figure 10C), and EFEMP1 (*r* = 0.72) presented a strong correlation with serum creatinine level (Figure 10F). Thus, the expression alteration of EFEMP1 may contribute to the occurrence and progression of DN. Furthermore, we conducted correlation analysis using samples from multiple kidney-related diseases. Characteristic genes remained significantly correlated and the trends were consistent with that of DN samples (Figures 10G–I), suggesting that the characteristic genes may play a critical role in the progression of kidney diseases.

## 4. Discussion

Diabetic nephropathy is a complex chronic disease and could eventually lead to the development of renal failure. Therefore, identifying the predictors of DN based on the pathogenesis of DN is of great significance for the prevention and early intervention of DN. In recent years, there has been an increasing interest in the role of aging in the pathogenesis of DN. This study used bioinformatics to explore aging-related characteristic genes in DN. We used RF and SVM-RFE algorithms to construct models for screening, and the two methods were contracted to obtain three intersecting genes: EFEMP1, GHR, and VEGFA. The ROC results showed good DN diagnostic performance of the three genes in the test cohort, and they showed consistent expression patterns in the glomerular test cohort. Therefore, we proposed that EFEMP1, GHR and VEGFA are potential aging-related characteristic genes for DN. These three characteristic genes can be considered DN diagnostic biomarkers and used to develop therapeutic targets.

There are four isoforms of GHR, of which GHRd3 is mainly expressed in the kidney, bladder, adrenal glands and brainstem. The GHR signaling pathway plays an essential role in cell growth, metabolism, cell cycle control and immunity through the JAK/STAT and SRC pathways. The glomerular podocyte is involved in the composition of the glomerular filtration barrier and provides epithelial coverage for the capillaries. It has been suggested that podocyte depletion is considered to be a marker of glomerulosclerosis (40). Previous studies have shown that podocytes express GHR, which induces Notch signaling in response to growth hormone (GH), and sustained activation of Notch signaling leads to non-productive cytoplasmic division and mitosis-induced cell death (41). GH induces TGF-β1 signaling and provokes cell cycle reentry of otherwise quiescent podocytes, and inhibiting the activation of TGF-smad signal is beneficial to DN (42, 43). In addition, an animal test has shown that mice overexpressing GH develop glomerular hypertrophy, Albuminuria and glomerulosclerosis (44), while termination of GHR signaling could control GH expression (45). A recent study shows that GHR deletion, JAK2 and insulin-like growth factor(IGF-β) inhibition abort GH-induced nephrogenic malformations (46). In training and test cohorts, GHR expression was low in the glomerulus of DN patients and upregulated in the renal tubules. Few studies have elucidated the metabolic mechanisms of GHR in the kidneys of DN patients. In our opinion, the down-regulation of GHR expression in the glomerulus may be related to the negative feedback regulation of GHR by DN. DN leads to hypertrophy and even failure of podocytes, which in turn causes a feedback reduction in GHR expression to inhibit GH damage to the glomerulus and tubules. However, further verification is needed about how GHR acts in the kidneys of DN patients.

Vascular endothelial growth factor A is a critical endogenous vascular growth factor, mainly expressed in glomerular podocytes and renal tubular epithelial cells (47). It is a crucial mediator in promoting angiogenesis and vascular remodeling and an essential substance in maintaining the dynamic homeostasis of the glomerular filtration barrier (48). It has been suggested that a reduction in VEGFA leads to impairment of the glomerular filtration barrier, proteinuria and renal dysfunction (49). Clinical studies have shown that in the early stages of diabetic nephropathy, VEGFA levels are elevated in the urinary vessels of diabetic patients (50). Moreover, there is a clinical correlation between urinary vascular VEGFA levels and the extent of DN lesions (51). In a diabetic mouse model, Veron et al. (49) found that the knockdown of VEGFA resulted in acute renal failure and albuminuria associated with endothelial hyperplasia, thylakoid lysis and microaneurysms. More Evidence indicates Worsening Proteinuria and Glomerular Microangiopathy in Patients Treated with Anti-VEGFA (52, 53). In the pathology of diabetic nephropathy, renal fibrosis is considered a complex and irreversible process in the advanced stages of diabetic nephropathy (54). VEGFA, as a growth factor essential for angiogenesis, inhibits the expression of Smad3 and miR192, thereby suppressing IGF-β-induced endothelial interstitial transformation and ameliorating renal fibrosis (55).

EFEMP1 is an extracellular matrix protein involved in cell structure and signaling, promoting vascular endothelial growth factor expression. In training and test cohorts of DN, EFEMP1 was lowly expressed in both glomerular and tubular tissues. EFEMP1-inactivated mice exhibit reduced reproductive capacity and an earlier phenotype associated with aging (56). EFEMP1 encodes an extracellular protein called fibuin-3, which is involved in extracellular matrix remodeling and cell proliferation (57). It has been suggested that fibuin-3 plays a vital role in maintaining the integrity of connective tissue and regulating aging (56). In exploring the pathogenesis of osteoarthritis, Hasegawa et al. (58) found that Fibulin3 governs the differentiation of adult progenitor cells and is reduced in expression in aging and osteoarthritis. More than one study has pointed out that EFEMP1-encoded fibuin-3 is highly expressed in vascular endothelial and epithelial cells and is particularly abundant in small vessels (59, 60). At the same time, the glomerulus, one of the significant lesion tissues in DN, consists mainly of capillary endothelial cells, glomerular basement membrane and epithelial cells. Therefore, the expression of EFEMP1 may be downregulated when pathological changes occur in DN.

Senescent cells can secrete various pro-inflammatory and chemokines, and we further explored differences in immune infiltration between the DN samples and controls. According to the ssGSEA algorithm, most immune cells are highly expressed in DN. Many studies have shown that immune cell infiltration plays a vital role in the pathogenesis of DN. Moon et al. (16) concluded that the kidney T cell (CD4, CD8) and B cell infiltration were significantly increased in the kidneys of DN patients, and the increase of CD4 T cells and CD20 T cells was positively correlated with albuminuria. Smith et al. (61) concluded that aberrant recruitment and activation of T cells in the mesenchyme is an underlying pathological mechanism of diabetic nephropathy, and B cells also promote the progression of diabetic nephropathy. Wang et al. (62) showed that macrophages were associated with diabetic kidney injury, and DN was more severe in mice with an increased M1 phenotype of macrophages. Zheng et al. (63) observed accumulation and degranulation of mast cells in the interstitial periglomerular, peritubular and perivascular regions in renal biopsies in type 2 diabetic patients. In this study, the association between EFEMP1, GHR and VEGFA and cellular immune infiltration was further explored. Our results showed that three characteristic genes were negatively correlated with most immune cells with statistical significance. A study reveals that lowered GH/IGF1 activity promote inflammatory activity, causing long term tissue damage and systemic chronic inflammation (64). GHR has a strong expression in B-cells, while T-cells and natural killer cells exhibit considerably lower levels of GHR expression (65). Another study shows that exposure of aged hematopoietic stem cells to recombinant growth hormone restores B-cell output (66). Ishikawa et al. (67) find that the induction of GH is critical for suppressing innate immune cells such as NK cells/NK T cells and macrophage-mediated apoptosis. Moreover, it has been suggested that VEGFA can promote the accumulation of circulating MDSCs by activating the JAK2-STAT3 pathway, which can inhibit effector T cells (68–70). Increased VEGF expression can lead to immune suppression *via* the inhibition of dendritic cell maturation and the reduction of T-cell infiltration (71). A study shows that genetic inactivation of VEGFA improves clearance of senescent cells by natural killer cells (72), and a negative correlation is observed between a particular B-cell and VEGF levels (73). Research on the mechanism of EFEMP1 and immune cells is deficient, and our work provides direction for further studies.

Additionally, we use the TarBase repository to predict miRNAs associated with the characteristic genes. The three characteristic genes are connected by multiple shared miRNAs, and the modification of the shared miRNAs may be critical for the synergistic expression of the characteristic genes. Studies have shown that miR-15a-5p can regulate acute kidney injury induced by sepsis (74), and overexpression of miR-23b-3p can induce apoptosis and autophagy (75). Zang (76) and Xie et al. (77) have found that miR-23b-3p and miR-15a-5p are up-regulated in DN patients and sequentially hypothesize that miR-23b-3p and miR-15a-5p can be miRNA markers of DN. miR-205-5p can promote cell proliferation, migration and anabolism, and inhibit inflammation (19). miR-195-5p is proven to promote intestinal epithelial recovery and attenuate the inflammatory response (78), and some have said that miR-20a-5p and miR-195-5p can improve ischemic kidney injury (79, 80). Godwin (81) and Zhang et al. (18) have found that miR-199a-3p is negatively associated with albuminuria in DN patients and that miR-199a-3p can inhibit high glucose-induced inflammation and apoptosis by regulating IKKβ/NF-κB signaling pathway in renal tubular epithelial cells. miR-126 can prevent microvascular dysfunction and improve inflammatory outcomes (82). Therefore, the shared miRNAs may have a meaningful impact on the mechanical process of co-expression of GHR, VEGFA and EFEMP1, and they may participate in the pathogenesis and development of DN together with these three characteristic genes. Besides, we also established mRNA-TF networks using potential transcription factors. We found that GATA2 has a regulatory effect on GHR and VEGFA. The expression of GATA2 activates the gene regulatory network of hemangioblast and induces the formation of hemangioblast through the activation of VEGFA ligand (83, 84), and VEGFA is important for maintaining the integrity of the glomerular filtration barrier as well as a survival factor for the podocyte (85). The mechanism of GATA2 regulation of GHR has not been elucidated, but we speculate that GATA2 may play a regulatory role in GHR in terms of cell production. It has been suggested that GATA2 mediates the number of cytokines (86), while GHR plays an important role in cell growth and metabolism as well.

There may be some limitations in our study. Firstly, the sample used for analysis and validation was relatively small, which may lead to a lack of reliability in the results. Secondly, this study is based on publicly available data and lacks validation experiments. Further basic and prospective research would be beneficial for the prevention and treatment of DN, and the mechanism between the characteristic genes and immune infiltration in DN remains to be investigated and validated in further studies.

## 5. Conclusion

In this study, GHR, VEGFA and EFEMP1 were identified as potential biomarkers for diagnosing and treating diabetic nephropathy. This finding provides clues to the mechanisms of disease development in DN at the transcriptome level. In addition, we evaluated the immune infiltration landscape of the characteristic genes and explored their molecular regulatory mechanisms. These explorations may provide insights into the management and treatment of patients with diabetic nephropathy.

## Data availability statement

The survey data of this study publicly available in the GEO and Nephroseq V5 database, and the names of the repository/repositories and accession number(s) can be found in the article/Supplementary material.

## Author contributions

YL designed the study. ZL collected research data. YL and JH performed data analysis. MJ, DL, and PZ wrote the manuscript. ZF, XH, and HL revised the manuscript. All authors contributed to the article and approved the submitted version.

## Funding

This work was supported by National Natural Science Foundation of China (grant number: 82274419) and Natural Science Foundation of Guangdong (grant number: 2020A1515010775).

## Conflict of interest

The authors declare that the research was conducted in the absence of any commercial or financial relationships that could be construed as a potential conflict of interest.

## Publisher’s note

All claims expressed in this article are solely those of the authors and do not necessarily represent those of their affiliated organizations, or those of the publisher, the editors and the reviewers. Any product that may be evaluated in this article, or claim that may be made by its manufacturer, is not guaranteed or endorsed by the publisher.

## Footnotes

^1^ https://www.ncbi.nlm.nih.gov/geo/

^2^ https://genomics.senescence.info/

^3^ https://www.gsea-msigdb.org/gsea/msigdb/index.jsp

^4^ https://cn.string-db.org/

^5^ https://www.networkanalyst.ca/

^6^ http://v5.nephroseq.org/
