# Supplementary figures and images for: Identification and validation of aging-related gene signatures and their immune landscape in diabetic nephropathy
Source: Front Med (Lausanne). 2023 Jun 19;10:1158166. doi: 10.3389/fmed.2023.1158166 (PMC10316791; doi:10.3389/fmed.2023.1158166)

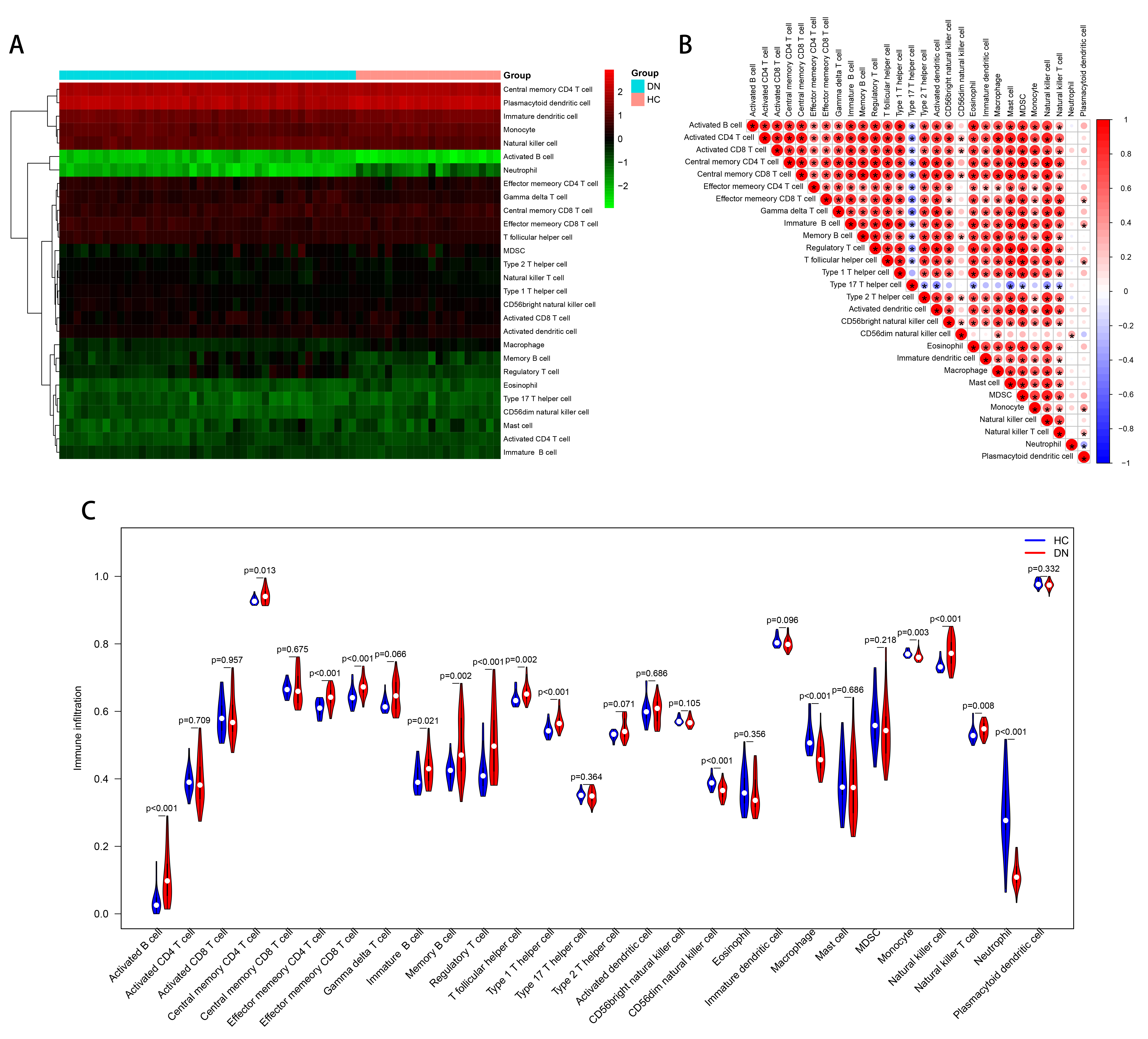

Supplement: Supplementary file 1 [file Image_1.JPEG]
